# Role of AI in the analysis of total knee arthroplasty

**DOI:** 10.6026/973206300213725

**Published:** 2025-10-31

**Authors:** Anuraag Mohanty, Priyankar Nanda, Preethiv Rajendran

**Affiliations:** 1 Department of Orthopedics, Kalinga Institute of Medical Sciences, KIIT, Bhubaneswar, Odisha, India

**Keywords:** Total knee replacement, Artificial intelligence, ChatGPT, KOOS score, peri-prosthetic joint infection, outcome prediction, machine learning, ROC analysis

## Abstract

This retrospective cohort assessed a ChatGPT-based AI model for 1-year KOOS prediction and infection risk in 98 primary total knee replacement (TKR) patients. The model predicted based on preoperative clinical, demographic, and intra-operative data. The model under predicted the mean knee injury and osteoarthritis Outcome Score (KOOS) by 7-8 points compared to surgeon-reported outcomes (p = 0.02) with moderate correlation (r = 0.45).Predicted risk of infection (2.4%) was nominally higher than observed (1.8%), with an ROC-AUC of 0.70. Directionally accurate, the model requires further fine-tuning prior to clinic use.

## Background:

With proven improvements in joint function, pain management, and quality of life, total knee replacement (TKR) is widely accepted as the most effective treatment for end-stage knee osteoarthritis [1]. After surgery, functional status and subjective healing are frequently assessed using patient-reported outcome measures (PROMs), such as the Knee Injury and Osteoarthritis Outcome Score (KOOS) [2]. Accurate pre-operative prediction of post-operative outcomes is important for maximizing patient education, setting realistic expectations, and guiding clinical decision-making on the basis of a variety of factors, including demographic data, co-morbidities, radiological assessment, and intra-operative data [3]. Traditional regression and machine learning techniques have already been utilized to a great extent to make post-TKR predictions [4]. Generative language models have, more recently, been demonstrated as an alternative, where heterogeneous clinical data can be combined into an overall predictive model [5]. We used, in this study, an AI system based on ChatGPT to predict pre-operative and intra-operative data. Therefore, it is of interest to report its performance in predicting 1-year KOOS scores and the risk of post-TKR infection.

## Materials and Methods:

## Study design and population:

This study was conducted as a retrospective observational study in the Department of Orthopedics, Kalinga Institute of Medical Sciences, Bhubaneswar, where 98 consecutive patients who had undergone primary TKR for osteoarthritis from January 2023 to January 2024 were included. Postoperative follow-up of at least 1-year was available for all the patients. Inclusion Criteria includes individuals who have been diagnosed with primary knee osteoarthritis underwent primary TKR with Documented surgeon-reported KOOS and infection status at 1-year follow-up. Revised TKR or cases with secondary arthritis (e.g., post-traumatic) with incomplete medical records or loss to follow-up are excluded. Data were retrospectively extracted from the hospital's electronic medical records (EMR) and comprised the following parameters: Demographic and clinical parameters includes.

## Demographics:

Body mass index (BMI), age, and gender.

## Preoperative functional status:

KOOS score (with an average of approximately 52.1 ± 11.4) and visual analogue pain score (VAS).

## Clinical examination:

Assessment of knee range of motion (flexion and extension), measurement of fixed flexion deformity, quantification of varus/valgus deformity (in degrees), and grading of ligamentous stability on a standardized scale.

## Radiological parameters taken:

## Alignment measurements:

Hip-Knee-Ankle (HKA) angle; varus alignment is indicated by negative values.

## Angular measurements:

Medial Proximal Tibial Angle (MPTA) and Mechanical Lateral Distal Femoral Angle (mLDFA).

## Slope and patellar metrics:

Posterior tibial slope and patellar tilt angle.

## Osteoarthritis severity:

Kellgren-Lawrence grade determined from standard weight-bearing radiographs.

## Laboratory parameters:

Preoperative values including C-reactive protein (CRP), erythrocyte sedimentation rate (ESR), hemoglobin, and serum albumin.

## Intra-operative parameters:

Intraoperative findings such as soft-tissue balancing, requirement for ligamentous release, and final implant alignment as determined by intraoperative fluoroscopy.

## Post-operative outcome measures:

Primary Outcome: Surgeon-reported 1-year KOOS score. Secondary Outcome: Occurrence of postoperative periprosthetic joint infection within 1 year.

## AI predictive modeling:

For each patient, a standardized data package was compiled and provided to our AI model.

The prompt included: Based on the following patient data:

[1] Age, gender, and BMI

[2] Comorbidities (e.g., diabetes, hypertension, smoking status)

[3] Preoperative KOOS and VAS scores

[4] Radiological parameters: HKA angle, mLDFA, MPTA, posterior tibial slope, patellar tilt, and Kellgren-Lawrence grade

[5] Clinical examination: knee range of motion, fixed flexion deformity, varus/valgus deformity (in degrees), and ligament stability

[6] Laboratory values: CRP, ESR, hemoglobin, and albumin

[7] Intraoperative details: operative time, blood loss, soft-tissue balancing, and final implant alignment

## Please predict:

[1] The 1-year postoperative KOOS score, and

[2] The risk of postoperative infection (expressed as a percentage probability).

Our AI source for this study was ChatGPT-a generative language model that utilizes advanced natural language processing techniques. Although initially developed for general-purpose language tasks, ChatGPT's extensive training on a broad corpus of medical literature enables it to integrate multifaceted clinical data similarly to regression-based predictive models.

## Example case analysis:

A representative patient from our cohort was a 65-year-old female with a BMI of 30, controlled hypertension, and no history of diabetes or smoking. Her preoperative KOOS score was 42, and her VAS pain score was 7/10. Radiologically, her hip-knee-ankle (HKA) angle measured -5° (indicating mild varus alignment), and she was assigned a Kellgren-Lawrence grade III. On clinical examination, her knee exhibited a range of motion from 0 to 110° with a 5° fixed flexion deformity, and ligamentous stability was noted as intact. Laboratory values were within normal limits. Intra-operatively, the procedure was uneventful with an operative time of 90 minutes and minimal blood loss; final implant alignment was deemed satisfactory. For this patient, our AI-based model predicted a 1-year KOOS score of approximately 74 and an infection risk of 1.4%. The corresponding surgeon-reported outcomes were a KOOS score of 77 and no postoperative infection. This example underscores that while the model captures the overall trend in clinical recovery, it tends to underestimate the magnitude of improvement.

## Statistical analysis:

To conduct statistical studies, IBM SPSS version 26 was used. Categorical variables were represented by frequencies and percentages, whereas continuous variables were represented by means ± standard deviations. AI-predicted KOOS scores and surgeon-reported scores were compared using a paired t-test; a p-value < 0.05 was deemed statistically significant. The linear link between the two sets of KOOS scores was evaluated using Pearson's correlation coefficient. The area under the curve (AUC) was calculated using receiver operating characteristic (ROC) curve analysis for infection risk prediction; an AUC of 0.70 was deemed indicative of acceptable discriminative performance.

## Results and Discussion:

The study cohort included 98 patients who underwent primary TKR at the Kalinga Institute of Medical Sciences, Department of Orthopedics. The mean age was 66 ± 8.3 years, and the mean BMI was 29.8 ± 4.5 kg/m^2^. Females comprised 61.7% of the cohort. The average preoperative KOOS score was 52.1 ± 11.4, consistent with values typically observed in patients with advanced knee osteoarthritis. Table 1 (see PDF) presents demographic and clinical parameters of the study cohort (n = 98), including mean age, mean body mass index (BMI), sex distribution, preoperative KOOS scores, and minimum follow-up duration. The AI model under-predicted functional recovery by approximately 7-8 points compared with surgeon-reported KOOS scores (p = 0.02). Pearson's correlation coefficient (r = 0.45, p < 0.001) demonstrates a moderate positive correlation, suggesting partial agreement between predicted and actual outcomes but highlighting missing intra-operative variables (Table 2 - see PDF). The observed PJI rate was 1.8%, while the AI estimated an infection risk of 2.4%. Receiver operating characteristic (ROC) curve analysis yielded an AUC of 0.70 (95% CI: 0.60-0.80), reflecting acceptable discriminative ability for infection prediction but underscoring the need for further model optimization (Table 3 - see PDF).

## KOOS score prediction:

Surgeon-Reported KOOS: The average 1-year KOOS score recorded by the surgeon was 76.3 ± 14.5.

## AI-Predicted KOOS:

The AI model predicted an average 1-year KOOS score of 68.5 ± 16.2. A paired t-test demonstrated a statistically significant mean difference of -7.8 points (p = 0.02), indicating that the model's predictions under predicted functional recovery by approximately 7-8 points.

## Correlation Analysis:

Pearson's correlation coefficient between AI-predicted KOOS scores and surgeon-reported scores was 0.45 (p < 0.001), reflecting a moderate positive correlation. This suggests that although there is a statistically significant association, the model does not capture all the variability inherent in clinical outcomes.

## Infection risk prediction:

## Actual Infection Rate:

The observed postoperative peri-prosthetic infection rate in the cohort was 1.8%.

## AI-Predicted Infection Risk:

The AI estimated an overall peri-prosthetic infection risk of 2.4% ROC curve analysis for infection risk prediction produced an AUC of 0.70 (95% CI: 0.60-0.80), indicating that the model has acceptable discriminative ability to distinguish between patients who develop infections and those who do not. The AI-based predictions indicate that, while the model captures general trends in postoperative recovery, systematic discrepancies exist between predicted and actual outcomes. Patient expectations and satisfaction after TKR are influenced by baseline KOOS scores, The ratio of body mass index (BMI) to co-morbidities [6, 7]. In particular, a higher BMI has been repeatedly associated with poorer functional outcomes after TKR [8]. Additionally, the moderate Pearson correlation coefficient (r = 0.45) between AI-predicted and surgeon-reported KOOS scores suggests that certain variables-potentially including intra-operative factors such as soft-tissue balancing and implant alignment- may not be fully represented in the predictive algorithm [9, 10]. Contemporary research in outcome prediction has demonstrated the potential for advanced machine learning techniques to improve prognostic accuracy by training on extensive, domain-specific datasets [11]. In the context of infection risk, accurate stratification is crucial for mitigating peri-prosthetic joint infection- a complication that significantly impacts patient outcomes and healthcare resource allocation [12, 13]. The ROC analysis showing an AUC of 0.70 for infection risk prediction reflects acceptable discriminative performance but also highlights the need for further model optimization. The emerging literature underscores both the potential and current limitations of applying AI in clinical settings, suggesting that further refinement and specialized training of these models are necessary to enhance their predictive performance [14-15]. In this context, our study provides valuable insights into the application of an AI-based model using ChatGPT for outcome prediction in TKR while indicating avenues for future research.

The AI model achieved an area under the curve (AUC) of 0.70 (95% CI: 0.60-0.80), demonstrating acceptable discriminative ability to differentiate between patients who developed infections and those who did not. Figure 1 (see PDF) illustrates the balance between sensitivity and specificity in infection risk prediction using the AI-based algorithm. In Figure 2 (see PDF), a moderate positive Pearson correlation coefficient (r = 0.45) was observed, indicating that AI-based predictions capture general trends in postoperative recovery but may not fully reflect intra-operative factors such as implant alignment and soft-tissue balancing. This highlights both the potential and current limitations of applying AI for functional outcome prediction in TKR.

Recent literature reinforces the transformative role of AI and machine learning in orthopaedic surgery. Batailler *et al.* (2022) [16] reviewed the integration of AI in arthroplasty and emphasised its capacity to enhance diagnostic precision, optimise patient selection, and support personalised care pathways. Their work provides a foundation for the use of predictive algorithms in TKR, showing how advanced computational models can improve prognostic reliability beyond conventional risk scores. Building on this, Mickley *et al.* (2024) [17] presented a prospective validation of AI-based clinical decision tools in arthroplasty, reporting that predictive algorithms can reduce variability in outcome assessment and assist surgeons in preoperative planning. These advancements reflect a shift from descriptive clinical data analysis toward dynamic, real-time predictive modelling. In parallel, Rodríguez-Merchán (2022) [18] has highlighted the importance of long-term outcome prediction in TKR, noting that peri-prosthetic infection and functional decline remain critical challenges. The integration of AI into this domain addresses an unmet need by providing risk stratification models that can improve postoperative surveillance and guide targeted interventions. Our findings resonate with this perspective, showing that even with moderate discriminative power, AI systems can contribute to early identification of at-risk patients, thereby improving resource allocation and patient safety. Taken together, these advances illustrate how the field is moving toward evidence-based precision arthroplasty, where AI systems not only analyse historical data but also dynamically learn from diverse clinical variables. By demonstrating proof-of-concept performance, our study contributes to this trajectory of scientific progress. The results emphasise that while AI models such as ChatGPT cannot yet replace surgeon expertise, they represent an important adjunctive tool in the evolution of orthopaedic outcome prediction. Further refinement through domain-specific training, larger multicentric datasets, and integration of intra-operative data will be essential for achieving clinical-grade reliability.

## Conclusion:

This retrospective analysis tested a ChatGPT-based AI model for predicting 1-year KOOS scores and infection risk following primary TKR and achieved moderate correlation (r = 0.45) and fair discrimination (AUC = 0.70). The model underestimated functional outcome by 7-8 points and overestimated infection risk. Although promising, optimization and validation in larger prospective cohorts are required prior to clinical application.
